# Case Report: Eculizumab and ECMO Rescue Therapy of Severe ARDS in Goodpasture Syndrome

**DOI:** 10.3389/fmed.2021.720949

**Published:** 2021-09-23

**Authors:** Michael Sobotta, Onnen Moerer, Oliver Gross

**Affiliations:** ^1^Clinic of Anaesthesiology, University Medical Center Goettingen, Goettingen, Germany; ^2^Clinic of Nephrology and Rheumatology, University Medical Center Goettingen, Goettingen, Germany

**Keywords:** Goodpasture syndrome, lung failure, ARDS, ECMO-therapy, eculizumab, type IV collagen, anti-GBM disease, vasculitis

## Abstract

**Introduction:** Goodpasture's syndrome is a life-threatening autoimmune type IV collagen disease characterized by the presence anti–glomerular basement membrane antibodies, rapid progressive glomerulonephritis and/or pulmonary hemorrhage.

**Methods:** Here, we describe new therapeutic options, which take recent advances in unraveling Goodpasture's pathogenesis into account.

**Results:** In a 17-year old male, severe Goodpasture's syndrome resulted in acute respiratory distress syndrome (ARDS). Within 1 day after hospital admission, the patient required extracorporeal membrane oxygenation (ECMO). Despite steroid-pulse and plasmapheresis, ARDS further deteriorated. Eleven days after admission, the patient was in a pre-final stage. At last, we decided to block the complement-driven lung damage by Eculizumab. Three days after, lung-failure has stabilized in a way allowing us to initiate Cyclophosphamide-therapy. As mechanical ventilation further triggers Goodpasture-epitope exposure, the patient was taken from pressure support - breathing spontaneously by the help of maintaining ECMO therapy. After a total of 24 days, ECMO could be stopped and pulmonary function further recovered.

**Conclusions:** In conclusion, our findings suggest that life-threatening organ-damage in Goodpasture's syndrome can be halted by Eculizumab as well as by lung-protective early withdrawal from pressure support by the help of ECMO. Both therapeutic options serve as new tools in otherwise hopeless situations to prevent further organ-damage and to gain time until the established immunosuppressive therapy works in otherwise lethal autoimmune-diseases.

## Introduction

Patients with Goodpasture syndrome develop rapid progressive glomerulonephritis and lung hemorrhage. This life-threatening autoimmune disease is caused by circulating anti-glomerular basement membrane (GBM) autoantibodies, which produce linear IgG staining of glomerular capillaries in kidney biopsy specimens (1, 2). The α3(IV) NC1 domain has been identified as the Goodpasture autoantigen (3). Involvement of the lung and GBM is caused by the presence of this target antigen in these tissues. Multigenetic loci that influence the T-cell response, autoreactive CD4+ helper T cells, and macrophage activity modulate the disease (4, 5).

Two dominant α3(IV) NC1 epitopes have been described; they do not bind native cross-linked α3/4/5 hexamers until they are dissociated (6). The epitopes are cryptic and concealed by covalent bonds of the α3/4/5 hexamers. The formation of these sulfilimine crosslinks is catalyzed by peroxidasin with hypohalous acids as intermediary products (7). The presence of aggressive hypohalous acids in this chemical process may explain why, in the case of a failure of this process, such as in Goodpasture syndrome, fulminant organ damage occurs, with GBM ruptures, plasma leakage, crescent formation and pulmonary hemorrhage.

Extracorporeal membrane oxygenation (ECMO) is used to back up and bridge blood gas exchange in patients with severe acute respiratory distress syndrome (ARDS) refractory to conventional mechanical ventilation (8–10). ECMO helps gain time for the treatment of the lung disease and de-escalate the ventilation settings (11). One very rare cause of ARDS is Goodpasture syndrome. The use of ECMO in patients with Goodpasture syndrome has been reported in only a few cases (12–15).

Eculizumab is a terminal complement inhibitor that binds to the human C5 complement protein, thus blocking the generation of proinflammatory C5a and C5b-9. It is approved for the treatment of atypical hemolytic-uremic syndrome and paroxysmal nocturnal hemoglobinuria (16, 17). Eculizumab has been used in several complement driven severe diseases, such as refractory membranoproliferative glomerulonephritis (18); however, it has not previously been applied in Goodpasture syndrome. The scientific evidence on the possible role of complement (dysregulation) specific to Goodpasture syndrome is limited. In the kidney, immunofluorescence may demonstrate the presence of complement components, in particular C3 and C1q, along the GBM (2, 7).

We describe a patient who developed the most severe ARDS due to Goodpasture syndrome, thus requiring ECMO therapy. Despite steroid pulse therapy and plasmapheresis, no significant improvement in lung function was achieved. At a pre-final stage, we blocked the complement-driven lung damage using a single dose of Eculizumab. In addition, early weaning from pressure support ventilation with the help of maximal ECMO therapy further stabilized the patient in this critical situation. With the maintenance therapy of Cyclophosphamide and Prednisolone, as well as intermittent plasmapheresis, the pulmonary function recovered within 3 weeks. These findings indicate that in addition to the early withdrawal of pressure support ventilation bridged by ECMO, Eculizumab may slow down complement-driven lung damage in life-threatening autoimmune diseases, thus gaining time until the immunosuppressive and immune-reductive therapies, including Cyclophosphamide and plasmapheresis, can take effect.

## Methodology

Written informed consent was provided by the patient and both parents for the publication of his case. All methods, procedures and laboratory test described in the results section are documented in the electronic patient record of our intensive care unit and origin from the routine methods and documentation system used in our University hospital Goettingen.

## Results

### Clinical Presentation

We report a 17-year-old male smoker in excellent physical condition (soccer player) who contacted his family physician because of mild dyspnea and cough. The family physician made the diagnosis of bacterial bronchitis and prescribed azithromycin. The patient continued his apprenticeship in the scaffolding industry, including the use of spray cans (with equipment for respiratory protection). Despite antibiotics, the symptoms became worse, and aggravating hemoptysis appeared. After 6 days, the patient contacted his family doctor again with dyspnea and hemoptysis and was admitted to a local city hospital. The patient was immediately transferred to the ICU and required intubation and mechanical ventilation within several hours. The diagnosis of ambulant acquired pneumonia was made, and antibiotic therapy was escalated to clarithromycin and meropenem. The same day, due to complete failure of the conventional mechanical ventilation, the city hospital contacted the ECMO task force of our University hospital. The team performed veno-venous cannulation on site, and the patient was transported to our University hospital by helicopter. In summary, the 17-year old patient required intubation on the first day of hospital admission and also, a few hours later, ECMO therapy needed to be initiated on the first day of hospital admission.

At the time of admission to our University hospital, the ECMO blood flow was 4.5 l/min, and the oxygen flow of 3 liters was accompanied by continued invasive ventilation at a bi-level positive airway pressure mode (BIPAP) with a positive end-expiratory pressure (PEEP) of 15 cmH2O, a positive inspiratory pressure (PIP) of 30 cmH_2_0 and a fraction of inspired oxygen (FiO_2_) of 80%.

**Figure 1:**
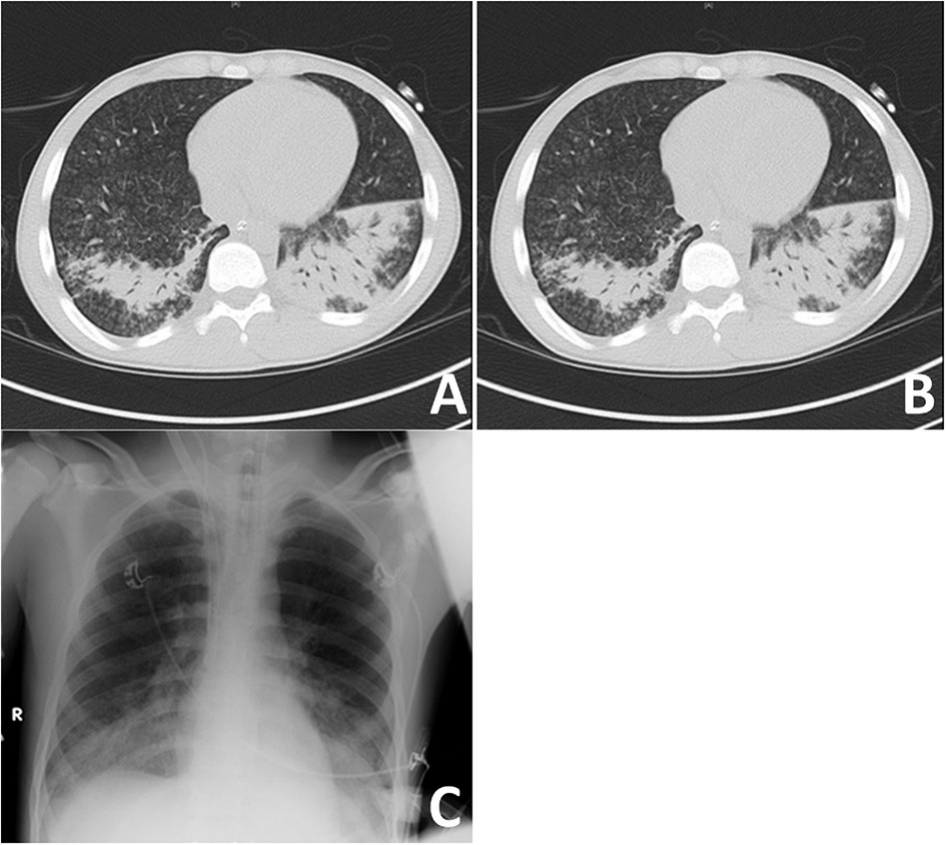
Imaging of most severe autoimmune lung disease on admission. **(A)** Recruitment CT-scan for acute respiratory distress syndrome (ARDS) at FiO_2_ of 100% in deep analgo-sedation, as well as muscle relaxation with cis-atracurium at a PEEP-level of 5 cmH_2_O and PIP at 15 cmH_2_O with tidal volumes at 6 ml/kg ideal body weight (IBW) in an expiratory hold after 3 min at this level; **(B)** CT-scan after 1 min of recruitment at a PEEP-level of 10 cm H_2_0 and a PIP of 35 cm H_2_O, followed by 1 min at a PEEP-level of 20 cm H_2_O and a PIP of 45 cm H_2_O at an inspiratory hold; **(C)** Conventional imaging with bilateral inflammatory consolidations in lower lobes.

A recruitment CT scan for acute respiratory distress syndrome (ARDS) did not show a significant recruitment potential of the lungs (Figures 1A,B). Small airway disease was diagnosed with bilateral inflammatory consolidations in the lower lobes, which, at this time, appeared as a typical bilateral pneumonia in the conventional im-aging (Figure 1C). Bronchoscopy showed rather pale mucous membranes, no swelling and no pus; however, fresh blood and blood clots of different ages, as well as very vulnerable airways were present. Bronchoalveolar lavage was taken for microbiologic workup; however, lung biopsy was not performed because of very stringent anticoagulation under ECMO therapy. Due to the complete absence of kidney involvement besides microhematuria without acanthocytes (Table 1), Goodpasture syndrome was not suspected during the first days at our ICU. Antibiotic therapy was further escalated to meropenem, vancomycin and clarithromycin (Table 1).

**Table 1:** Urine analysis, laboratory tests and microbiological diagnostic workup on admission.

**Urine test Values**	**Result**	**Reference**
Urine erythrocytes (sediment)	>20	Neg
Urine acanthocytes (sediment)	neg	Neg
Urine dysmorphic erythrocytes (sediment)	neg	Neg
Urine protein [mg/l]	225.6	10–140
Urine immunoglobulin G [mg/l]	neg	Neg
Urine Alpha1-microglobuline [mg/l]	16.40	<2.0
Urine Albumin g/g Creatinine	31.91	<30
**Blood test Values**	**Result**	**Reference**
Hemoglobin [g/dl]	8.7	13.5–17.5
White blood cell count [tsd/μl]	15.4	4.0–11.0
Platelet count [tsd/μl]	303	150–350
Creatinine [mg/dl]	0.77	0.62–1.08
Blood urea nitrogen [mg/dl]	18	7–21
Procalcitonin [μg/l]	17.1	< = 0.1
Interleukin 6 [pg/l]	98.5	<7.0
C3c [g/l]	0.56	0.83–1.52
C4 [g/l]	0.11	0.13–0.37
Lactate-dehydrogenase [U/l]	323	105–235
Microbiological workup	Testing for	Results
Blood cultures	*Bacteria*	Negative
Tracheal secretion cultures	Bacteria	Negative
Tracheal secretion PCR 1	Influenza A/B, RSV, EBV, CMV, HSV, VZV	Negative
Tracheal secretion PCR 1	Adenovirus-DNA	Positive
Tracheal secretion PCR 2	Influenza A/B, RSV, EBV, CMV, HSV, VZV	Negative
Tracheal secretion PCR 2	Adenovirus-DNA	Negative
Serology 1	Aspergillosis, Candidosis, Legionellosis, Chlamydia, Mycoplasma pneumoniae and Coxiella	Negative
Serology 2	Influenza A/B, Adenovirus	Negative
Serology INF-y-Tbc	M. Tuberculosis	Negative
Bronchoalveolar lavage 1	Bacteria, M. Tuberculosis	Negative
Bronchoalveolar lavage 2 PCR	Adenovirus, Influenza A/B, RSV, EBV, CMV, Pneumocystis jerovieci, Mycoplasma pneumoniae, Chlamydia trachomatis and pneumoniae, VZV	Negative
Bronchoalveolar lavage 3 culture	Candida dubliniensis	Positive
Urine culture	Bacteria	Negative

Three days after admission, the microbiological examinations showed no significant results with the exception of one positive finding of adenovirus-DNA and Candida dubliniensis in a culture from the bronchoalveolar lavage (Table 1). Both findings were not suspected to be the cause of the severe ARDS. This finding led to further examination, including a positive result for antinuclear antibodies (ANA-immunofluorescence 1:320; cANCA and pANCA negative). The patient's worsening critical condition led us to initiate Prednisolone pulse therapy at day four after admission to our ICU. The proof of anti-glomerular basement membrane (anti-GBM) antibodies and low complement levels (C3c and C4, see Table 1) soon led to the diagnosis of Goodpasture syndrome (without kidney involvement) as the cause of severe ARDS.

### Treatment of Goodpasture Autoimmune Disease

Daily plasmapheresis was initiated; however, Cyclophosphamide therapy had to be postponed due to the high Procalcitonin levels and the very critical lung condition of the patient, which would not have tolerated worsening of the possibly ongoing bacterial pneumonia.

Despite daily plasmapheresis and steroid pulse therapy, the respiratory condition further deteriorated to a prefinal condition at the ninth day after admission. As an ultima ratio, we applied an off-label single dose rescue therapy with 900 mg eculizumab (plasmapheresis was paused for 48 h; meningococcal prophylaxis and vaccination were applied). Consequently, within the next 3 days, lung failure stabilized in a way that enabled us to initiate intravenous Cyclophosphamide therapy at bi-weekly doses of 500 mg. In parallel, Prednisolone was reduced to 1 mg/kg per day, and daily plasmapheresis was continued until anti-GBM antibodies could no longer be detected in ELISA.

**Figure 2:**
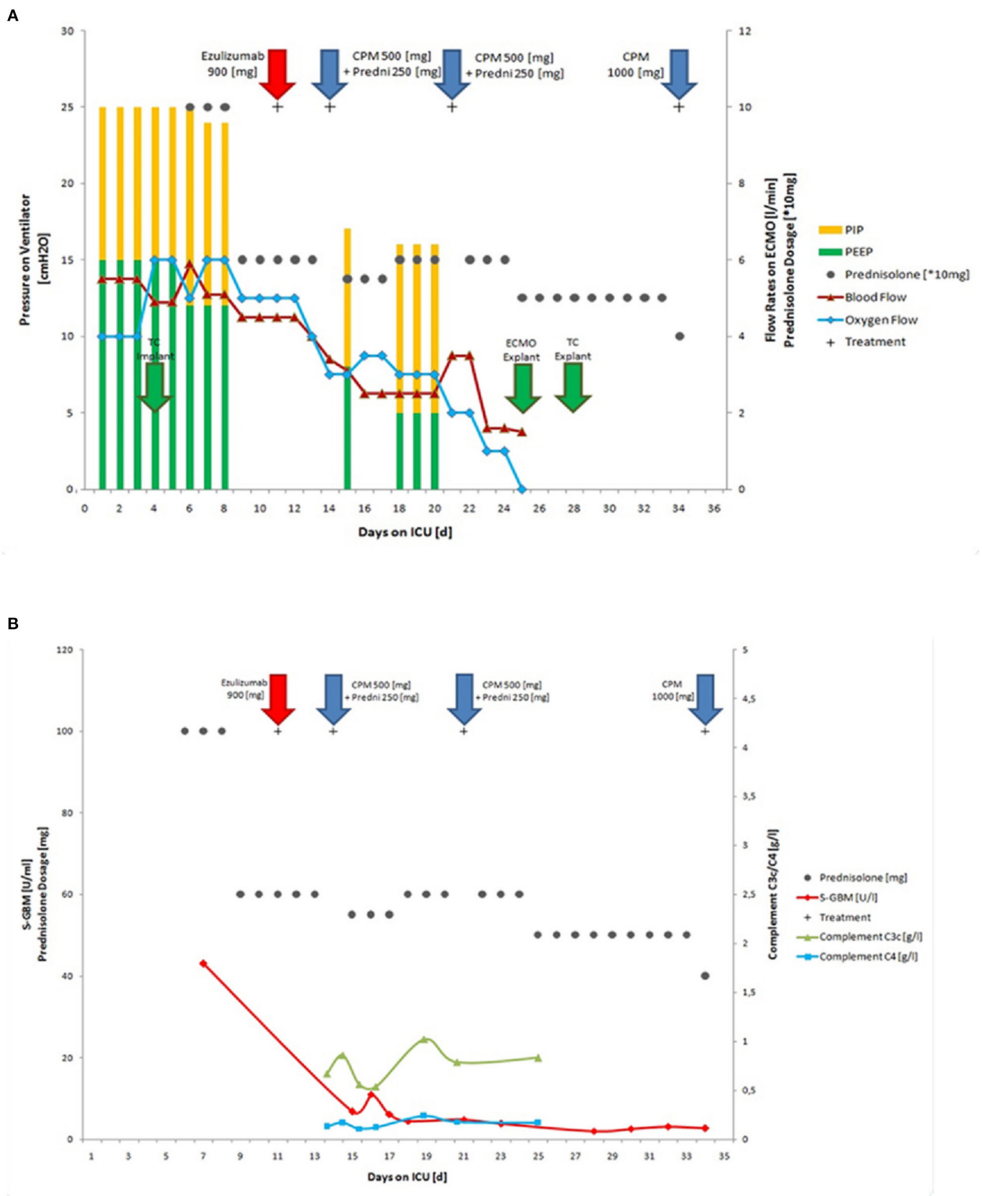
Course of autoimmune disease during therapy. The 17-year old patient required intubation on the first day of hospital admission and also, a few hours later, ECMO therapy needed to be initiated on the first day of hospital admission. **(A)** ECMO and mechanical ventilation conditions during treatment in the ICU, including implantation and explantation of tracheal cannula (TC explant), PIP, PEEP, blood flow, oxygen flow and immunosuppressive therapy with Eculizumab, Cyclophosphamide, and Prednisolone; **(B)** Titers of anti-GBM and complement C3/C4 during therapy (normal ranges: S-GBM Elisa <7 U/ml; C3 0.83–1.52 g/l; C4 0.13–0.37 g/l) ECMO, extracorporeal membrane oxygenation; TC, tracheal cannula; CPM, Cyclophosphamide; Predni, Prednisolone; PIP, positive inspiratory pressure; PEEP, positive end expiratory pressure; Explant, explantation.

As mechanical ventilation triggers Goodpasture epitope exposure, we aggressively reduced pressure support; using dexmedetomidine, an adequate awakening without significant signs of delirium could be established. No neurological failures were identified. The tidal volumes further improved under intermittent CPAP ventilation. After only 3 days, 17 days after admission, the mechanical ventilation weaning was accomplished. The patient was removed from pressure support and was breathing spontaneously bridged by maximal ECMO therapy. At this time, the ECMO conditions included a blood flow of 4.5 l/min and an oxygen flow of 5 l/min. ECMO-weaning was successful at day 24 after admission to our University hospital (Figure 2A). Full mobility was reached after 34 days without subjective dyspnea during normal physical stress. After the last course of Cyclophosphamide (1,000 mg), the patient was subsequently discharged from our ICU unit and transferred back home to his local hospital (Figure 2B). There, he received a final 500 mg dose of Cyclophosphamide, and the tracheostoma was closed. Anti-GBM antibodies were monitored on a weekly basis for 6 months and remained negative. Prednisolone was tapered to 5 mg and discontinued after 6 months. All kidney tests remained normal, and lung function completely recovered. Four months after admission to our ICU, the patient scored his first goal in a full 90 min soccer game in his youth team and now plays and trains regularly for an adult team 12 months after.

## Discussion

Although approved for atypical hemolytic-uremic syndrome and paroxysmal nocturnal hemoglobinuria (7, 8), the use of Eculizumab has been described in other complement driven severe diseases, such as refractory membranoproliferative glomerulonephritis (18). However, to our knowledge and according to the manufacturer (personal information by pharmacovigilance Alexion Pharmaceuticals, Munich, Germany), it has not previously been applied in Goodpasture syndrome. For that reason, both parents have been informed about the off-label use of Eculizumab prior application and have given their written consent. Our example might contribute to expand the (limited) therapeutic options in very severe Goodpasture cases.

The inhalation of hydrocarbons or tobacco may trigger Goodpasture syndrome for unknown reasons. Bromine is a required cofactor for peroxidasin, which is the only currently known essential function of bromine in animals (6, 19). In our patient, the inhalation of hydrocarbons may have affected the reactant bromide, thus indirectly influencing peroxidasin production of aggressive hypohalous acids. In addition, it may have altered the patient's extracellular chloride gradient, which is required for type IV collagen assembly (20). This toxic cocktail may have ultimately led to the uncovering of the cryptic NC1α3(IV) epitope and initiated the Goodpasture syndrome disease process. In conclusion, aggressive hypohalous acids and further ventilation pressure trauma resulted in the most severe, life-threatening lung hemorrhage. A severe bacterial lung infection was considered a further initial trigger. As a consequence, severe systemic bacterial infection (as indicated by high procalcitonin levels) in a very critical respiratory situation initially hindered us from applying cyclophosphamide in addition to steroid pulse therapy and plasmapheresis.

The diagnosis of Goodpasture syndrome was confirmed via its clinical presentation, the presence of anti-GBM antibodies in ELISA and immunofluorescence, complement depletion and its clinical and serological response to immune-suppressive therapy. Diagnosis was not further confirmed by kidney biopsy as the kidney involvement was minimal without proteinuria or hematuria. Moreover, despite initial bronchoscopic evaluation, severe lung hemorrhage and strict anti-coagulation therapy during ECMO therapy hindered us from performing lung (or kidney) biopsy. The response to therapy was monitored by respiratory parameters and anti-GBM titers.

Although lymphocytes and macrophages contribute generally to pulmonary and renal damage in Goodpasture syndrome, autoreactive T cells play a unique role in the progress of the disease: they not only enhance B cell function and antibody production, but a distinct T-cell epitope may have a direct causative function in glomerular and alveolar injury (2, 4). The T-cell epitope is similar to some microbial peptides; therefore, distinct pulmonary infections (as in our patient) may not only damage the basement membrane but also provoke an autoimmune response. After the initiation of Good-pasture syndrome, activation of the complement cascade and proteases further contribute to pulmonary and GBM damage. Complement in our patient is not considered to be critical for his Goodpasture's pathogenesis, but to be a very important enhancing pathway for additional lung injury. Consequently, our rationale underlying the application of the complement inhibitor Eculizumab was to prevent further generation of proinflammatory C5a and also the lytic membrane pore C5b-9, which are thought to cause major lung damage. Despite the novel use of Eculizumab, the clinical improvement subsequently observed may have also been a delayed response to conventional components of therapy (plasmapheresis and steroids).

In addition, the mechanical stress on the lung tissue by pressure support ventilation is thought to have further triggered antigen presentation and therefore maintained epitope exposure in this life-threating autoimmune disease. Thus, decreasing mechanical strain on the alveolar epithelium via modification of the ventilation parameters might have contributed to the patient's recovery. Here, additional damage to the epithelial cells complement autoregulation via surface bound regulators might have been prevented or reduced. In our patient, a fulminant progress led to severe ARDS according to the Berlin definition (21), thus requiring ECMO therapy, which can significantly decrease the mortality (22). In our patient, we selected a lung-protective ventilation strategy with the lowest tidal volumes. Low tidal volumes have been reported to reduce further lung injury and mortality, as well as the number of ventilator-days (in other lung diseases) (23, 24). To further support lung recovery, we performed an early wake up on ECMO. Similar strategies have previously been reported in other settings, including the bridging to lung transplantation (25). In contrast to previously reported cases (12–15), we aimed to wean the patient off of mechanical ventilation prior to weaning him from ECMO therapy, taking into account the mechanical triggers and pathogenesis of Goodpasture syndrome (26, 27). The combination of complement blockade to prevent further lung damage and early weaning from pressure support ventilation with the help of prolonged ECMO led to a rapid improvement of the previous almost desperate situation: the titer of anti-GBM antibodies decreased within days, and the weaning from the respirator was accomplished 7 days after eculizumab therapy. The ECMO weaning was accomplished after an additional 7 days, 24 days after admission and 14 days after eculizumab.

In conclusion, our findings suggest that Eculizumab may heal, irrespective of the trigger, complement-driven organ damage in life-threatening Goodpasture syndrome. Furthermore, lung-protective early withdrawal from pressure support bridged by ECMO may prevent further harm to the lung tissue in Goodpasture syndrome. Both therapeutic options take Goodpasture pathogenesis into account and may serve as an important tool in otherwise hopeless situations to prevent further organ damage and gain time until the established immunosuppressive therapy begins to take effect in otherwise fatal autoimmune diseases.

## Data Availability Statement

The original contributions presented in the study are included in the article/supplementary material, further inquiries can be directed to the corresponding author/s.

## Ethics Statement

Ethical review and approval was not required for the study on human participants in accordance with the local legislation and institutional requirements. Written informed consent to participate in this study was provided by the participants' legal guardian/next of kin. Written informed consent was obtained from the minor(s)' legal guardian/next of kin for the publication of any potentially identifiable images or data included in this article.

## Author Contributions

MS, OM, and OG: conceptualization, methodology, and investigation. MS: writing—original draft preparation and visualization. OM and OG: writing—review, editing, and supervision. All authors contributed to the article and approved the submitted version.

## Conflict of Interest

The authors declare that the research was conducted in the absence of any commercial or financial relationships that could be construed as a potential conflict of interest.

## Publisher's Note

All claims expressed in this article are solely those of the authors and do not necessarily represent those of their affiliated organizations, or those of the publisher, the editors and the reviewers. Any product that may be evaluated in this article, or claim that may be made by its manufacturer, is not guaranteed or endorsed by the publisher.
